# Influence of long and short arms of X chromosome on maxillary molar crown morphology

**DOI:** 10.1371/journal.pone.0207070

**Published:** 2018-11-15

**Authors:** Mitsuko Nakayama, Osamu Kondo, Paula Pesonen, Lassi Alvesalo, Raija Lähdesmäki

**Affiliations:** 1 Oral Development and Orthodontics, Research Unit of Oral Health Sciences, Medical Faculty, University of Oulu, Oulu, Finland; 2 Medical Research Center Oulu, Oulu University Hospital, Oulu, Finland; 3 Department of Anatomy I, Nihon University, School of Dentistry at Matsudo, Matsudo, Japan; 4 Department of Biological Science, Graduate School of Science, University of Tokyo, Tokyo, Japan; 5 Northern Finland Birth Cohorts, Faculty of Medicine, University of Oulu, Oulu, Finland; Texas A&M University College of Dentistry, UNITED STATES

## Abstract

Although genes on the human X chromosome reportedly influence tooth crown morphology, little is known about X chromosome activation or inactivation systems relevant to morphological variations. We assessed the relationships between tooth crown size and crown morphological traits in females with Turner syndrome, the variants of which include complete absence of one X chromosome, lack of the short arm (Xp), or duplication of the long arms (Xq), and then estimated the functions of Xp and Xq in the process of unilateral X chromosome inactivation during tooth crown development. The mesiodistal and buccolingual diameters in the maxillary first (M1) and second (M2) permanent molars were compared among X chromosome karyotypes by multiple regression analyses, and their relationships with the development of Carabelli’s cusp and the distolingual cusp were analyzed using logistic regression analysis.

The crown sizes increased in the order of the 46,X,i(Xq) karyotype, 45,X and 45,X/46,XX karyotypes, and control group. A lower frequency of Carabelli’s cusp and higher frequency of a reduced distolingual cusp in M1 were characteristics of Turner syndrome. The overall M1 and M2 crown sizes differed among the X chromosome karyotypes, whereas a smaller crown size was associated with a reduced distolingual cusp but not with Carabelli’s cusp. Considering the differences in chromosome arrangement among females with Turner syndrome and the process of unilateral X chromosome inactivation, the observed results can be considered in terms of quantity or number of promoters/inhibitors during tooth crown development.

The X chromosome karyotypes have a strong influence on the overall crown sizes of the M1 and M2 molars because those karyotypes with variable numbers of active gene regions directly influence tooth germ development in an early stage of human odontogenesis. The later forming cusps, such as the distolingual cusp and Carabelli’s cusp, may be affected by this developmental prerequisite.

## Introduction

An increase or decrease in the number of human X chromosomes has various impacts on physical phenotypes in females. This has been clarified by previous studies of X chromosome aberrations, such as Turner syndrome (TS). Females with TS reportedly have specific physical characteristics, including short stature and reduced tooth crown sizes [1, 2, 3].

TS affects approximately 1 in 2500 live-born female infants [4]. The most common karyotype is 45,X (50%), the second most common is 45,X/46,XX (20%), and the third most common is 46,X,i(Xq) (15%) [5]. Females with the 45,X/46,XX karyotype are mosaics, with both a 45,X cell line and a 46,XX cell line; in contrast, those with the 46,X,i(Xq) karyotype possess one normal X chromosome and an isochromosome with two long arms. Their physical characteristics are similar to those of females with the 45,X karyotype; e.g., short stature [4,6].

A lack of one X chromosome or any structural variation has been considered to influence the physical growth, especially stature, of females with TS. Individuals with TS generally have a normal amount of growth hormone, but a study of a Finnish population showed that their final height was clearly shorter than that of normal female individuals (average of 146.9 vs. 165.3 cm, respectively) [1, 7].

Other studies of craniofacial morphology have focused on the maxilla and mandible in females with the 45,X karyotype and have suggested that a lack of one X chromosome leads to hypo-growth of the mandible [8]; specifically, the maxilla is of normal length and palatal height, but it is narrowed in width [9]. In addition to altered craniofacial bone growth, TS results in reduced tooth crown diameters and simplified crown morphology in female patients with the 45,X karyotype [3, 10]. Previous investigations that focused on the tooth sizes of females with the 45,X/46,XX karyotype have shown that the mesiodistal (MD) diameters of the permanent teeth were smaller than those of normal females (46,XX) but that the buccolingual (BL) diameters were close to normal [2]. The permanent crown diameters are smaller in females with the 46,X,i(Xq) and 45,X karyotypes and normal females [11].

**Fig 1:**
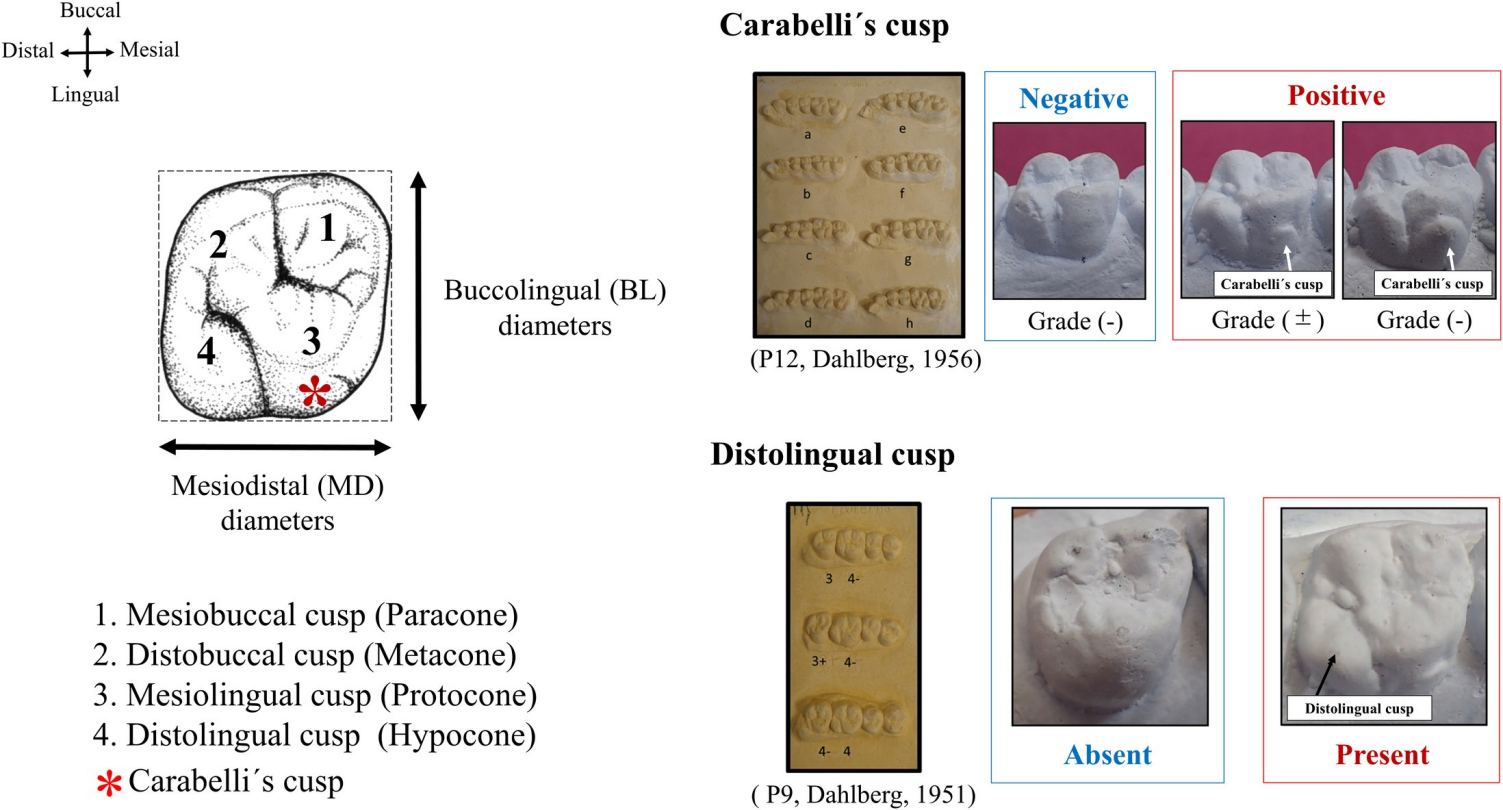
Tooth crown morphology in maxillary first permanent molars and classification of Carabelli’s cusp and the distolingual cusp.

In addition to size, sex chromosome aberration has also been assumed to be relevant to the crown shape of the maxillary permanent molars [12]. The appearance of secondary enamel knots is essential for normal formation of the future cusp in the early stage of molar development [13, 14, 15]. A normal four-cusp pattern is the most common in the maxillary first permanent molars (M1), whereas a three-cusp pattern frequently occurs in the second permanent molars (M2) (Fig 1). This variation results from the reduction of the distolingual cusp. An additional accessory tubercle called Carabelli’s cusp is expressed on the mesial side of the lingual surface of the maxillary molars [16] and may also contribute to a larger tooth crown size [17, 18].

The patterning cascade model (PCM) hypothesis takes the variation in size and shape of the molar crown into account [19]. The PCM predicts that in normal populations, the positions of early-developing cusps regulate the development of later-forming additional cusps. When the early cusps are closer to each other within a relatively larger tooth germ, which provides room for the later-developing cusp, then the later cusps are likely to be present and become larger in the outer free space [19]. In normal populations, the reduced intercusp distances on the maxillary deciduous and permanent molars significantly promote the expression of Carabelli’s cusp [20, 21]. In particular, the reduced distance between the mesiobuccal and mesiolingual cusps is regarded as an important factor for the expression of Carabelli’s cusp [22].

In their detailed description of the areas and volumes of the cusps on the maxillary first molar of females with the 45,X karyotype, Mayhall and Alvesalo [12] suggested that the later-developing cusps (i.e., the distobuccal and distolingual cusps) were more severely affected by the lack of one X chromosome than the early-developing cusps (i.e., the mesiobuccal and mesiolingual cusps). In addition, the intercusp distance between the distobuccal and distolingual cusps on the maxillary first molar of females with the 45,X and 45,X/46,XX karyotypes was shorter than that of normal populations [12, 23]. The observable growth suppression in the later-developing cusp (distolingual cusp) in females with the 45,X and 45,X/46,XX karyotypes seems to have a different developmental mechanism from that in normal human odontogenesis.

Given this situation, we can ask the following question: How do the karyotype differences among females with TS produce variations in tooth crown morphology?

When we consider the expression of X chromosomal genes such as Xp and Xq, we must consider the impact of the X chromosome inactivation system in females [24]. In general, an abnormal X chromosome (i.e., i(Xq)) is preferentially inactivated [25], whereas about 15% of the X-linked genes escape from the X chromosome inactivation system [26]. These include genes in the pseudoautosomal regions, one of which (PAR1) reportedly leads to short stature and other skeletal anomalies in patients with TS and tall stature in female patients with the 47,XXX karyotype [27, 28, 29, 30]. After considering the effect of variable karyotypes among female patients with TS along with the associated gene expressions, we can postulate a genetic control scheme in the X chromosome during tooth development.

This study was performed to elucidate the morphological variation of the molar crown in females with TS, the variants of which include mosaicism of the X chromosome and an additional long arm (Xq) or the lack of one short arm (Xp) on the X chromosome. We also estimated the effect of the genetic component and hypothesized a genetic scheme for tooth development with consideration of X chromosome inactivation systems.

More specifically, the objectives were:

1. to compare the tooth crown morphologies and tooth crown diameters in the maxillary first (M1) and second (M2) permanent molars from the dental casts of females with the 46,X,i(Xq) and 45,X/46,XX karyotypes versus those of females with the 45,X karyotype and population controls and analyze the relationships between the development (reduction) of Carabelli’s cusp and the distolingual cusp.
2. to evaluate the effect of the X chromosome karyotypes on crown sizes and cusp morphologies by conducting a multiple regression analysis and logistic regression analysis.
3. to estimate the genetic expressions or functions of Xp and Xq during tooth crown development.

## Materials and methods

### Subjects

The data on TS were derived from dental casts belonging to the KVANTTI Research Project on sex chromosome abnormalities headed by Professor Lassi Alvesalo in Finland. The females with the 46,X,i(Xq) and 45,X/46,XX karyotypes in this study were living in various parts of Finland, and their karyotypes had previously been confirmed for medical reasons by cytogenetic testing of skin fibroblasts. The data of the females with the 45,X karyotype were obtained from our previous research [31, 32]. The population controls were women from a rural community on the island of Hailuoto in Finland, and their dental casts had been collected by Professor Lassi Alvesalo for his doctoral thesis [33]. The numbers of subjects, all of whom were of Finnish ancestry, and their age distributions are shown in S1 Table. All study materials are stored at the Medical Faculty, University of Oulu, Finland.

The Institutional Review Board of the Faculty of Medicine, University of Turku, Finland reviewed and approved the protocol for informing the females with TS (KVANTTI material) and the population controls (Hailuoto material) about the study. All examinations were carried out after individual agreements had been made.

### Methods

The MD and BL crown diameters were measured using a sliding digital caliper (NTD12-15; Mitutoyo, Sakado, Japan) to an accuracy of 0.01 mm (Fig 1). The crown area [34] and reduction index [35] were also calculated. The crown area comprises the occlusal surface (i.e., crown area = MD diameter × BL diameter). The reduction index is the ratio of the crown diameters or crown area between M1 and M2 (i.e., reduction index (MD) = [MD of M2 / MD of M1] × 100, reduction index (BL) = [BL of M2 / BL of M1] × 100, and reduction index (crown area) = [crown area of M2 / crown area of M1] × 100). The expression of Carabelli’s cusp and development (reduction) of the distolingual cusp in M1 and M2 were observed using Dahlberg’s reference plaque [36, 37] as follows. Dahlberg’s P12 for Carabelli’s cusp: grade (−), a smooth surface (Dahlberg’s a); grade (±), a pit or furrow (Dahlberg’s b, c, d, e); and grade (+), a slight protuberance or cusp (Dahlberg’s f, g, h) (Fig 1). Development of the distolingual cusp was distinguished using Dahlberg’s P9: a three-cusp pattern (Dahlberg’s 3+, 3) and a four-cusp pattern (Dahlberg’s 4-, 4). The reliability of the classifications of Carabelli’s cusp and development of the distolingual cusp as well as of the tooth crown measurements in general were assessed by means of a double determination approach [32]. There were no significant differences between the first and second classifications and the measurements of the crown diameters.

All observations and measurements were collected by M.N. and applied to the right side wherever possible. If a tooth on the right side was not available because of absence or dental caries, the corresponding tooth on the left side was used.

### Statistical analysis

One-way analysis of variance (ANOVA) and the post-hoc Tukey’s honestly significant difference (HSD) test were used to compare the mean tooth crown diameters (MD, BL), crown areas, and reduction indices between different karyotype groups. In addition, the Kruskal–Wallis test was conducted to compare the medians among groups.

Fisher’s exact test was used to assess differences in the frequencies of Carabelli’s cusp and the distolingual cusp (three- and four-cusped patterns). The χ^2^ test of independence was used to determine whether there was a significant relationship between the expression of Carabelli’s cusp and the distolingual cusp.

Correlation and multiple regression analyses were conducted to resolve the variation in tooth crown sizes into various potential predictors such as the expression of Carabelli’s cusp and the distolingual cusp and the X chromosome karyotypes. The four karyotypes were synthesized into a model formula as three dummy variables. In addition, multiple logistic regression analysis was prepared to assess the associations of the expression of Carabelli’s cusp or the distolingual cusp with potential predictors of tooth crown sizes and X chromosome karyotypes. Finally, to take the effect of candidate genes in Xp and Xq into consideration, we used an average number of hypothetical promoters or inhibitors in Xp and Xq. When we consider the gene expression in the midst of Xp and Xq after unilateral X chromosome inactivation, the number should be 1 in all the karyotypes. When we hypothesize that the candidate genes exist in the pseudoautosomal regions, the number is variable. Thus, we assumed the number of candidate genes as follows: two in PAR1 and two in PAR2 for normal females (46,XX), one in PAR1 and one in PAR2 for females with the 45,X karyotype, and one in PAR1 and three in PAR2 for females with the 46,X,i(Xq) karyotype. The number for mosaic females with the 45,X/46,XX karyotype was set at a median of 1.5 in PAR1 and PAR2, respectively.

IBM SPSS Statistics Version 24 (IBM Corp., Armonk, NY) was used for the data analysis. The results were considered statistically significant when the p-value was ≤0.05.

## Results

### Comparisons of tooth crown sizes and morphology

The MD and BL crown diameters and the crown area (MD × BL) in both M1 and M2 were smallest in females with the 46,X,i(Xq) karyotype, followed by females with the 45,X and 45,X/46,XX karyotypes and finally the population controls, with one-way ANOVA and the Kruskal–Wallis test showing statistically significant differences (Table 1). Comparisons among the females with the 46,X,i(Xq), 45,X/46,XX, and 45,X karyotypes (among the TS groups) showed that the crown sizes of females with the 46,X,i(Xq) karyotype were always smaller than those of females with the 45X/46,XX and 45,X karyotypes. Tukey’s HSD test revealed that more pairwise comparisons were significant in M1 than in M2, although a significant difference was exclusively found in the comparison between population controls and females with TS (S2 Table).

**Table 1:** Basic statistics of the tooth crown diameters (mm), crown area (mm^2^), tooth shape, and reduction indices in the study groups.

		Karyotype	n	Mean	SD	Median	One-way ANOVA (df = 3)	Kruskal-Wallis test
							*P-value*^*a*^	*P-value*^*b*^
M1	MD diameter	46,X,i(Xq)	6	9.29	0.41	9.36	**< 0.001**	**< 0.001**
		45,X	81	9.66	0.58	9.60		
		45,X/46,XX	14	9.51	0.78	9.49		
		46,XX	140	10.52	0.66	10.45		
	BL diameter	46,X,i(Xq)	6	10.50	0.38	10.61	**< 0.001**	**< 0.001**
		45,X	82	10.57	0.50	10.47		
		45,X/46,XX	14	11.00	0.63	10.99		
		46,XX	140	10.99	0.60	11.01		
	Crown area (MD×BL)	46,X,i(Xq)	6	97.61	5.63	95.57	**< 0.001**	**< 0.001**
		45,X	81	102.24	9.46	101.21		
		45,X/46,XX	14	104.92	13.54	104.72		
		46,XX	139	115.90	11.85	116.70		
M2	MD diameter	46,X,i(Xq)	4	8.80	0.47	8.62	**< 0.001**	**< 0.001**
		45,X	56	9.10	0.55	9.10		
		45,X/46,XX	12	9.00	0.66	8.70		
		46,XX	70	9.49	0.57	9.42		
	BL diameter	46,X,i(Xq)	4	10.17	0.73	9.87	**0.047**	**0.001**
		45,X	54	10.50	0.69	10.46		
		45,X/46,XX	12	10.78	0.58	10.62		
		46,XX	75	10.78	0.67	10.84		
	Crown area (MD×BL)	46,X,i(Xq)	4	89.74	11.30	84.63	**0.001**	0.075
		45,X	54	95.44	10.10	95.11		
		45,X/46,XX	11	95.91	9.56	95.60		
		46,XX	71	102.47	10.67	102.68		
Reduction indices (M2/M1)	MD diameter	46,X,i(Xq)	4	95.07	3.74	95.20	**0.012**	**0.008**
		45,X	54	94.63	6.63	95.70		
		45,X/46,XX	12	95.02	4.64	95.01		
		46,XX	70	91.21	6.18	90.54		
	BL diameter	46,X,i(Xq)	4	97.40	4.91	96.85	0.952	0.953
		45,X	53	98.11	4.65	98.56		
		45,X/46,XX	12	98.26	4.93	97.86		
		46,XX	75	97.72	4.63	98.38		
	Crown area (MD×BL)	46,X,i(Xq)	4	92.57	5.40	90.40	0.092	0.106
		45,X	52	92.90	8.40	93.65		
		45,X/46,XX	12	93.40	6.96	93.20		
		46,XX	71	89.37	8.54	90.36		

SD, standard deviation; MD diameter, mesiodistal diameter; BL diameter, buccolingual diameter; Crown area, MD diameter × BL diameter.

^a^One-way analysis of variance and the

^b^Kruskal–Wallis test were used to assess differences in tooth crown size and reduction indices among females with Turner syndrome (46,X,i(Xq), 45,X, and 45,X/46,XX karyotypes) and population controls (46,XX karyotype).

The reduction indices (proportions of M2/M1) were similar in the TS group (Table 1), while the reduction indices of the MD diameter and crown area appeared to be larger in the TS than population control group, indicating a weaker M1 > M2 reduction in those diameters in the TS group. Tukey’s HSD test showed a significant difference only in the reduction index of MD between the females with the 45,X karyotype and the population controls (S2 Table).

**Fig 2:**
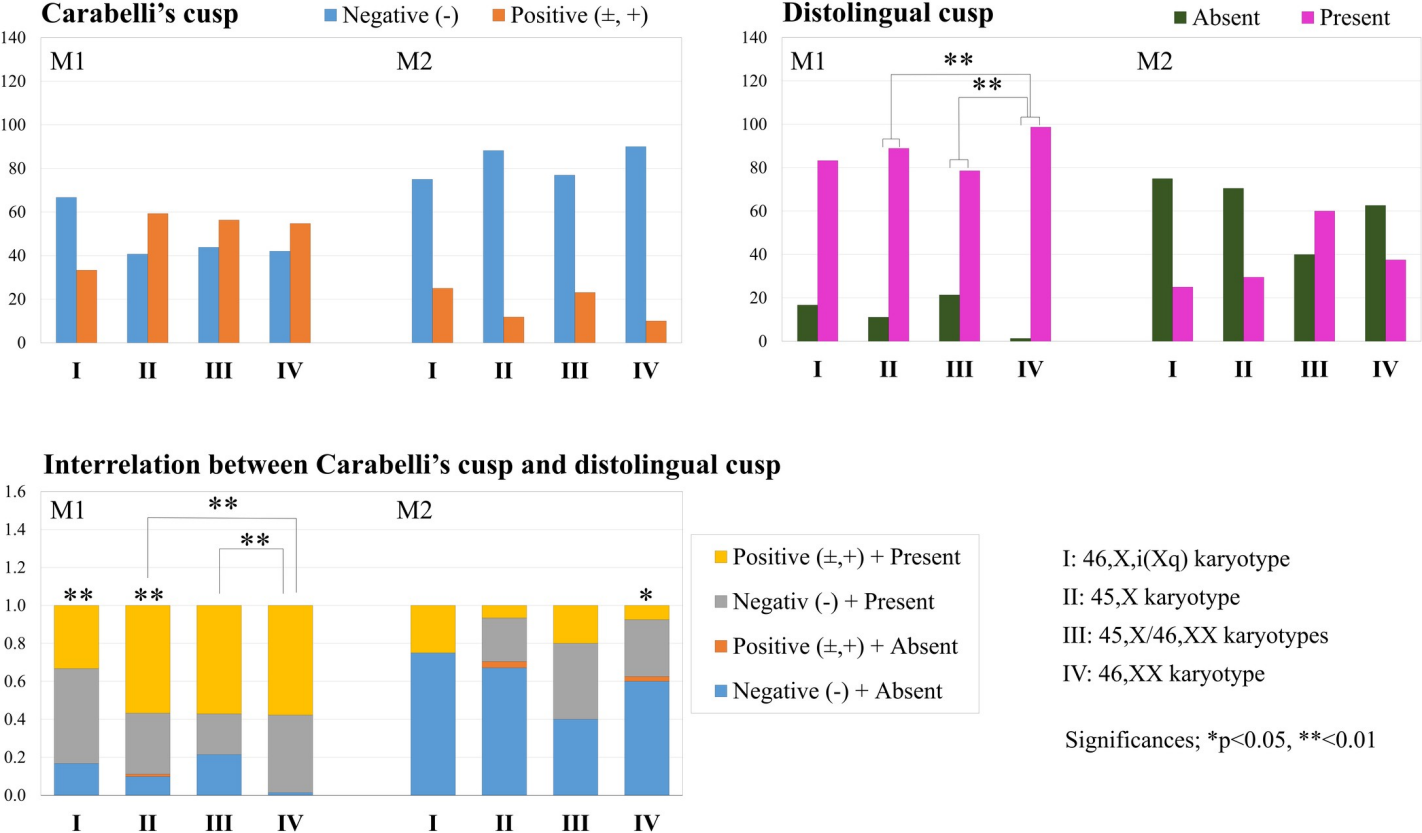
Frequency of Carabelli’s cusp and distolingual cusp and interrelation between two traits in the maxillary first (M1) and second (M2) permanent molars. Grades (±) and (+) were combined into one grade (±, +) for the analysis. Fisher’s exact test was used to assess the difference in the appearance of Carabelli’s cusp or the distolingual cusp. The χ^2^ test of independence was used to examine differences in the combination of Carabelli’s cusp and the distolingual cusp between groups.

The frequency of positive expression of Carabelli’s cusp (± and +) in M1 was lower in females with the 46,X,i(Xq) karyotype (33.3%) than in the other females with TS (45,X = 59.3%, 45,X/46,XX = 56.3%) and the population controls (58.0%), although there were no significant differences between the groups (Fig 2). Positive Carabelli’s cusp expression was seldom found in M2.

Females with TS showed 10% to 20% absence of distolingual cusp expression in M1 (46,X,i(Xq) = 16.7%, 45,X = 11.1%, and 45,X/46,XX = 21.4%), whereas almost all of the population controls showed positive expression (1.3% absent) (Fig 2). Appearance of the distolingual cusp was less frequent in M2 in general, without significant differences in the pairwise absent/present frequencies (Fig 2). The association between Carabelli’s cusp and the distolingual cusp was examined within and between karyotypes (Fig 2). In M1, the combination of negative expression of Carabelli’s cusp (−) and absence of the distolingual cusp (three cusps) occurred more frequently in females with TS (45,X/46,XX = 21.4%, 46,X,i(Xq) = 16.7%, and 45,X = 9.9%) than in the population controls (1.3%), although the χ^2^ test of independence showed significant differences between both females with the 45,X and 45,X/46,XX karyotypes and the population controls.

Absence of the distolingual cusp may have been linked to negative expression of Carabelli’s cusp in the TS group, but significantly skewed frequencies were only confirmed in females with the 45,X karyotype. In M2, no significant differences were found among the karyotypes, although significant skewness was found in the population controls.

### Relationship among tooth crown sizes, X chromosome karyotype, and crown morphology

The variation in the X chromosome karyotype had a consistent impact on the crown sizes in females with TS. A trend detected in all comparisons was smaller crown sizes in the TS than control group (46,XX), while the degree and pattern of size variation differed between variables (MD or BL diameter), tooth positions (M1 or M2), or associations of Carabelli’s cusp or the distolingual cusp.

**Fig 3:**
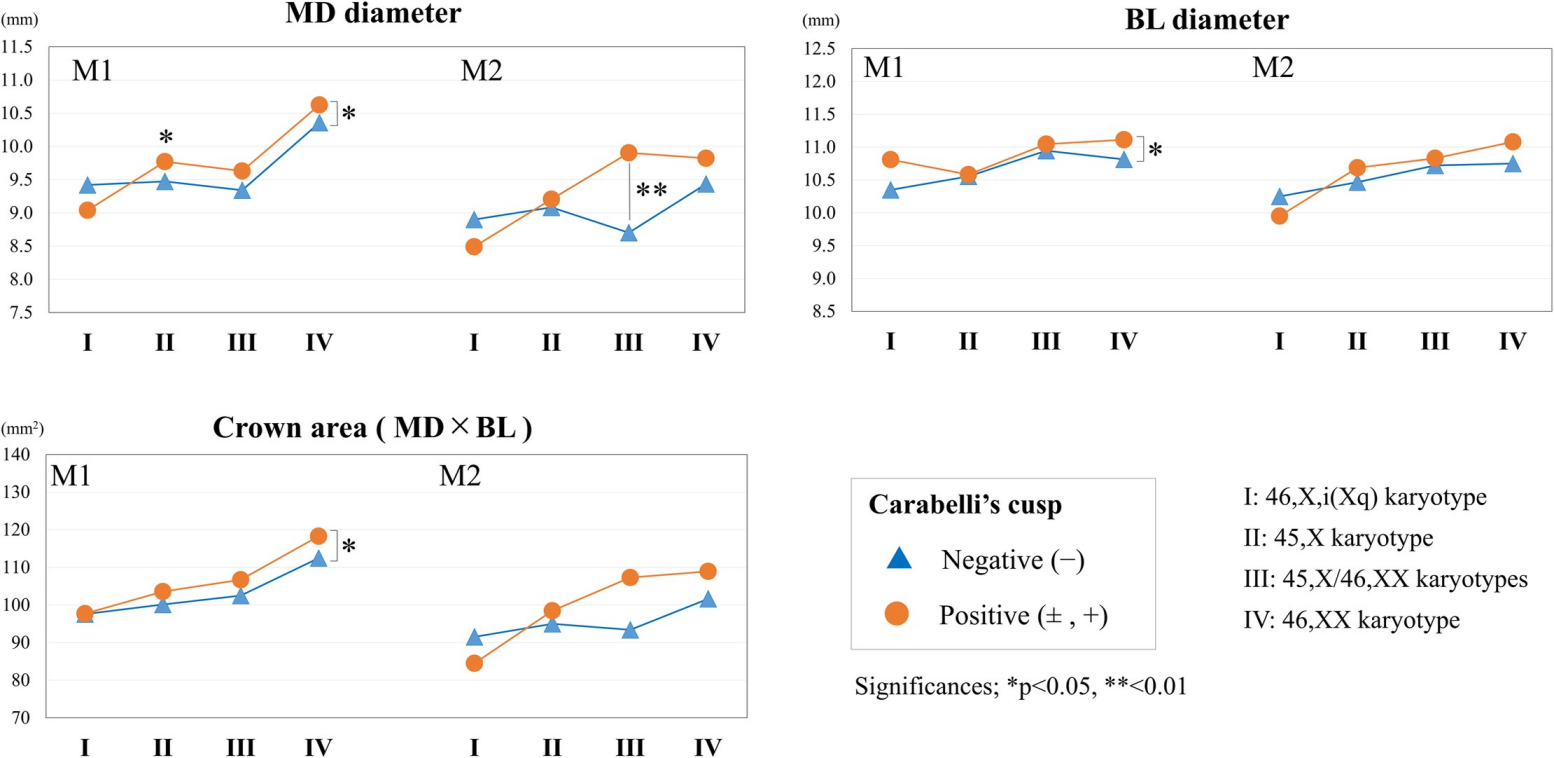
Mean tooth sizes (mesiodistal diameter, buccolingual diameter, crown area) and crown shape changes of Carabelli’s cusp expressions among females with Turner syndrome (46,X,i(Xq), 45,X/46,XX, and 45,X karyotypes) and population controls (46,XX karyotype). The t-test was used to assess the differences in tooth size and shape between negative and positive expression of the trait. *p < 0.05, **p < 0.01.

**Fig 4:**
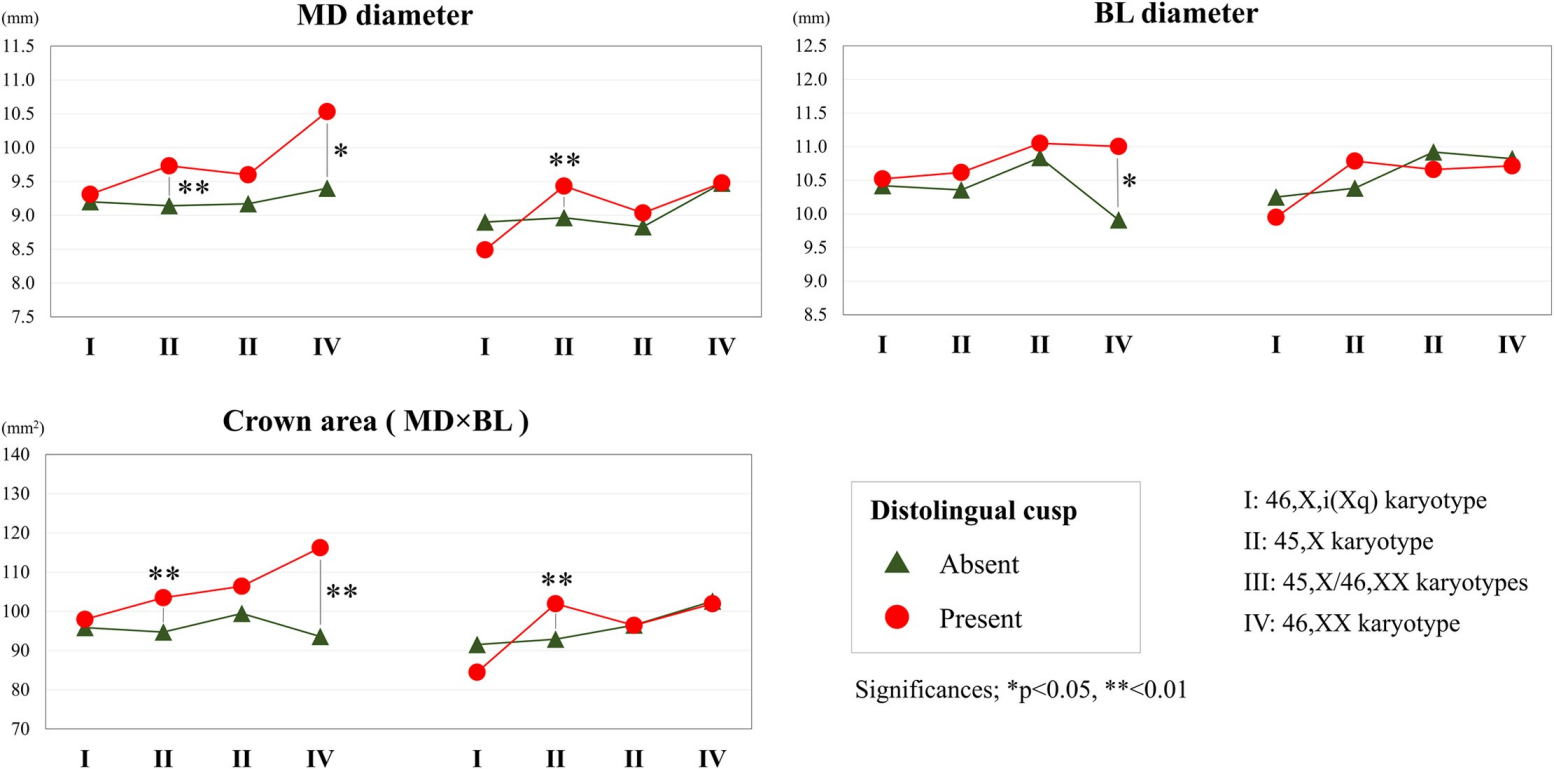
Mean tooth sizes (mesiodistal diameter, buccolingual diameter, crown area) and tooth shape changes of the distolingual cusp among females with Turner syndrome (46,X,i(Xq), 45,X/46,XX, and 45,X karyotypes) and population controls (46,XX karyotype). The t-test was used to assess the differences in tooth size between the absence and presence of the distolingual cusp. *p < 0.05, **p < 0.01.

Fig 3 shows the variability of crown sizes with the X chromosome karyotype and negative or positive expression of Carabelli’s cusp. Several measurements indicated significant differences between positive and negative expression of Carabelli’s cusp within the group. Fig 4 shows the crown size variations according to karyotype and distolingual cusp expression. The mean crown sizes of M1 with an absent distolingual cusp (three-cusped pattern) were roughly the same in all study groups (Fig 4). In contrast, M1 with the presence of a distolingual cusp (four-cusped pattern) showed obviously increases in size in the order of the 46,X,i(Xq) karyotype, 45,X- and 45,X/46,XX karyotypes, and population controls (Fig 4).

S3 Table shows the results of the multiple regression analysis among the tooth crown sizes, tooth crown morphologies, and X chromosome karyotypes. The karyotypes of both M1 and M2 exhibited positive and significant correlations with the crown sizes, and their adjusted regression coefficients were higher than those of the other predictor variables (S3 Table). When we consider the influence of karyotype differences on tooth crown sizes, the number of Xp and Xq as well as the number of X chromosomes presumably has a greater effect on the crown sizes in M1 and M2.

Based on these results, we next attempted to directly estimate the effect of the number of Xp (PAR1) and Xq (PAR2) in the multiple regression analysis (S4 Table). These results showed that the number of PAR1 had significant positive regression coefficients, indicating that increasing or decreasing M1 and M2 crown sizes are likely to be relevant to candidate genes in PAR1. In contrast, the number of PAR2 had negative coefficients for the M1 and M2 crown sizes without significant differences, indicating that plausible inhibitors are likely to exist in PAR2.

The results of the logistic regression analysis revealed that the expression of Carabelli’s cusp in M1 and M2 was associated with a higher odds ratio for the distolingual cusp than for any karyotypes or tooth crown diameters (S5 Table). Expression of the distolingual cusp was presumably related to the MD diameter as well as Carabelli’s cusp expression in M1, whereas this relationship seemed weaker in M2.

## Discussion

Previous investigations have indicated that X chromosome aberration has a significant impact on the tooth crown and root morphology in females. The additional X chromosome in females with the 47,XXX karyotype has a long-term effect of increased tooth crown sizes caused by the thicker enamel layer [38] and leads to enlargement of tooth root length or taurodontism [39, 40]. In contrast, the lack of one X chromosome leads to a reduced tooth crown accompanied by a thinner enamel layer and simplified morphology [3, 10, 41] as well as a short root length and variable shapes relative to normal populations [42, 43, 44, 45]. In the present study, the smallest tooth crown sizes and crown areas were recorded in females with the 46,X,i(Xq) karyotype, followed by the 45,X and 45,X/46,XX karyotypes and the population controls (Table 1). Thus, we should consider the observed variation among females with TS. The present results also indicate that the influence of the X chromosome aberration was stronger (indicating a greater degree of reduction) in M1 than M2.

These results coincide with previous studies based on KVANTTI material [2, 3, 12]. Townsend et al. [3] reported that the tooth crown diameters of early-developing teeth (e.g., the incisors and first molars) in females with the 45,X karyotype were strongly diminished relative to those of the later-developing teeth, and our previous comparison of M1 and M2 crowns also suggested a greater degree of reduction in M1 than M2 in females with the 45,X karyotype [32]. In the present analysis, the reduction indices of M2/M1 were almost the same in females with the 46,X,i(Xq) and 45,X/46,XX karyotypes as in those with the 45,X karyotype, and all were larger than those of normal females (Table 1). These findings indicate that the tooth crown size of early-developing molars (i.e., M1) in females with TS was reduced to a greater degree and more strongly influenced by X chromosome aberration than the later-developing molars (M2). This may fit the so-called inhibitory cascade model in which the first developing molar can inhibit the later-developing distal molars based on observations of sequential lower molar development in laboratory mice [46]. The model was also suitable to human incisors [47]. In more detail, our results showed an almost equal reduction rate of M1 > M2 among the TS groups, all of which lacked one Xp but possessed different numbers of Xq. We can therefore assume that the lack of one Xp first causes a reduction of the M1 crown sizes, and the later-developing molar (M2) has a more spacious field for crown development.

When we consider cusp development, the initiation or distribution of enamel knots is an essential factor for the future cusp and its form [13, 14, 15]. Mineralization of each cusp in the maxillary molars starts in the mesiobuccal cusp, followed by the mesiolingual, distobuccal, and distolingual cusps [48]. Finally, Carabelli’s cusp may develop as a possible fifth enamel knot in the human dentition, which may help to enlarge the final tooth crown sizes and modified phenotype [49].

In fact, the distolingual cusp was reduced in M1 of females with the 45,X karyotype; thus, the frequency of the three-cusp pattern was higher than in normal females [10, 32]. The same trend was confirmed in the other TS karyotypes in the present study (Fig 2). It seems that a reduced distolingual cusp in M1 is relevant in TS groups and may be caused by lack of one Xp.

Based on studies of the dentinoenamel junction, the distolingual cusp and Carabelli’s cusp are both derived from the same lingual cingulum region in normal populations [50]. Such a close link was not confirmed in the females with the 46,X,i(Xq) and 45,X/46,XX karyotypes of the present study (Fig 2), although our previous study revealed such a link in females with the 45,X karyotype [32]. This association seems to be specific to the 45,X karyotype; alternatively, the two traits may develop differently with respect to timing (early or late), thus exhibiting different influences on the tooth crown size.

Among another results, we recognized expression of the distolingual cusp as a plausible candidate factor that influenced the MD growth of molar tooth germ in the TS group (Fig 4; S3 and S4 Tables). The development of the distolingual cusp is reportedly relevant to the crown area in normal populations [51].

We found minor differences among the TS karyotypes in the present study (Table 1 and S2 Table). Some clinical reports have indicated that most females with the 45,X and 46,X,i(Xq) karyotypes have symptoms of primary ovarian insufficiency, whereas those with the 45,X/46,XX karyotype have a possibility of ovarian activity [52]. In addition, chronic thyroiditis (Hashimoto’s thyroiditis) is likely to develop in connection with TS, and the risk is higher in association with the 46,X,i(Xq) karyotype than the 45,X karyotype or other types of TS [6].

**Fig 5:**
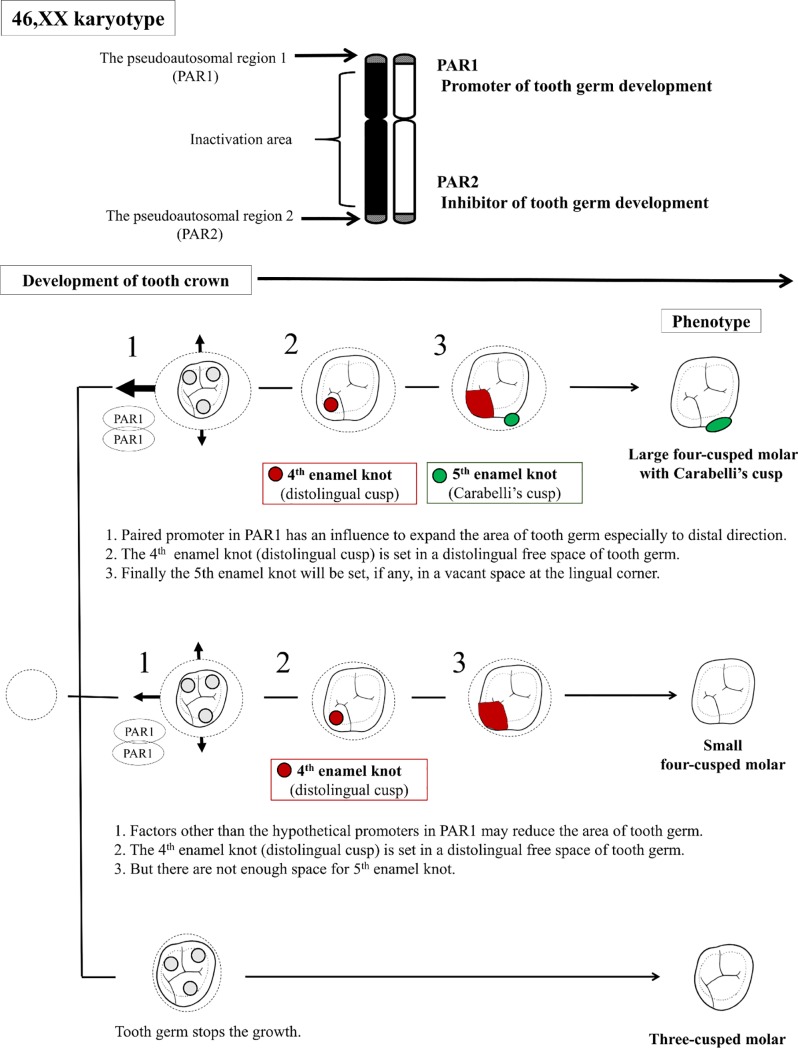
Proposed relationship between maxillary molar crown size and morphology in population controls (women with the 46,XX karyotype). This schema of estimation for women with the 46,XX karyotype was based on our results.

To integrate the observed phenotype differences in terms of gene expression patterns, we included the X chromosome inactivation system in females in the multiple regression analysis (S4 Table). The expression of this system in human cells can be observed from 12 days after fertilization [25]. About 15% of the X-linked genes in an X chromosome escape inactivation [26]. The pseudoautosomal regions (PAR1 and PAR2) are probably the most widely known regions that are not affected by X chromosome inactivation (Fig 5). PAR1 of Xp has attracted much attention as a container of the short stature homeobox (SHOX) gene because it contributes to short stature and other skeletal anomalies in patients with TS and to tall stature in females with the 47,XXX karyotype [27, 28, 29, 30]. This gene acts during early embryonic development to control the formation of the skeleton. Specifically, it plays a particularly important role in the growth and maturation of bones in the limbs, maxilla, and mandible [53]. These trends in stature seem to be comparable with those seen in tooth crown size when comparing females with TS versus the 47,XXX karyotype.

A recent study [54] using human embryonic stem cells suggested that lack of one copy of the colony-stimulating factor 2 receptor alpha (CSF2RA) gene in PAR1 may cause abnormal formation of the placenta in fetuses with the TS karyotype. As a result, most fetuses with the TS karyotype are spontaneously aborted during the early stage of pregnancy. It seems that the genes on Xp help to control the early stage of human embryo development.

**Fig 6:**
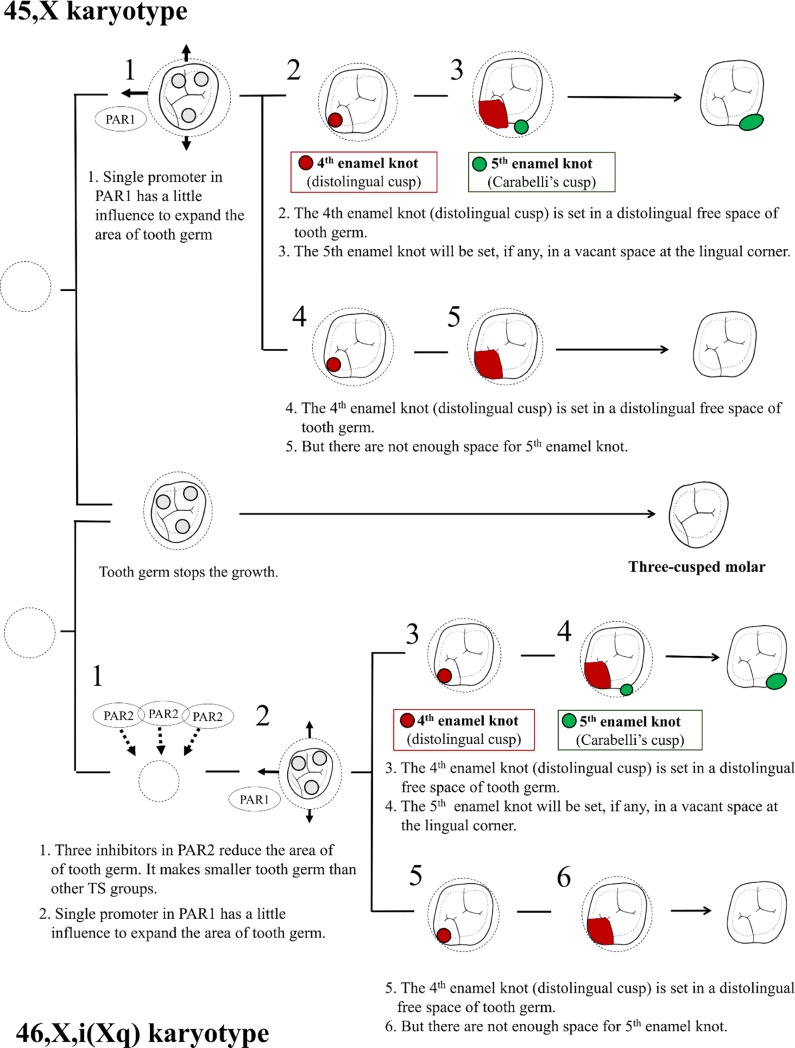
Proposed relationship between maxillary molar crown size and morphology in females with the 45,X and 46,X,i(Xq) karyotypes.

The results of the present study can be contextualized in a similar on-and-off activation scheme, like the SHOX gene. The hypothesis that a gene in PAR1 of Xp is the only promoter gene cannot explain the decrease in crown size among the TS karyotypes. We found that the crown sizes of M1 and M2 gradually decreased from the population controls to the females with the 45,X/46,XX, 45,X, and 46,X,i(Xq) karyotypes (Table 1). Therefore, we assume that an additional inhibitory gene for cell growth is present and formulated a hypothesis regarding the position of this inhibitor in PAR2 of Xq. Thus, three of these hypothesized genes are present in females with the 46,X,i(Xq) karyotype, which is greater than that in females with the 45,X/46,XX karyotype (one or two genes), 45,X karyotype (one gene), and population controls (two genes) (Figs 5 and 6). The above scenario may be consistent with the results regarding the distolingual cusp.

If we accept this hypothesis (i.e., that the candidate genes (promoters) for controlling tooth crown size are located in the active region (PAR1) in Xp in females whereas their inhibitor is located in the active region (PAR2) of Xq), then we will observe phenotype variation as a result of an interactive equilibrium between the promoters and inhibitors (Figs 5 and 6).

It is suggested that the paired promoter in Xp influences the expansion of the area of the tooth germ, especially in the distal direction (Fig 5). The fourth enamel knot (distolingual cusp) is set in a distolingual free space of the tooth germ. A reduced distolingual cusp (fourth enamel knot) in M1 is relevant in females with TS, which may appear at the same time as the effect of a reduced BL diameter (Table 1). After the fourth enamel knot occupies the distal corner in the tooth germ, the fifth enamel knot (Carabelli’s cusp) will finally be set, if any, in a vacant space at the lingual corner. The variation in Carabelli’s cusp may depend on the size of the free mesiolingual space that is not occupied by the fourth enamel knot.

In the present study, the differences in tooth crown phenotypes among females with TS seemed to be relevant to a combination of promoters and inhibitors; that is, the number of differences varies with TS karyotypes (Fig 6). These numbers of differences in candidate genes impact the tooth germ beginning in the early stage of human odontogenesis. In addition, the influence of these promoters and inhibitors can vary between the early-developing and later-developing molars (i.e., M1 and M2).

## Conclusions

The results of this study suggest that the X chromosome impacts the development of several particular tooth crown phenotypes. With the aim of clarifying the effect of X chromosomal aberrations on molar crown morphology, we observed the expression of Carabelli’s cusp and the distolingual cusp in the M1 and M2 permanent molars and investigated their relationship with tooth crown sizes (MD diameter, BL diameter, and crown area) in females with TS (i.e., females with the 46,X,i(Xq), 45,X, and 45,X/46,XX karyotypes). The smallest crown sizes were found in females with the 46,X,i(Xq) karyotype, followed by the 45,X and 45,X/46,XX karyotypes and finally the population controls. The size reduction in patients with TS occurred more in the MD diameter of M1. The lower frequency of positive expression of Carabelli’s cusp (not significant) and the higher frequency of the reduced distolingual cusp (three cusps) in M1 were characteristic of TS in contrast to the population controls.

The X chromosome karyotypes have a strong influence on the overall crown sizes of M1 and M2. The TS karyotypes vary in terms of the number of active regions in their short (Xp) and long (Xq) arms, which regulates the development of a tooth germ in the early stage of human odontogenesis. This may not influence later cusp development. Although the tooth crown sizes of females with TS are apparently associated with the expression of the distolingual cusp, the tooth germ size may be a prerequisite to the distolingual cusp. The expression of Carabelli’s cusp may be limited to a remnant area in the mesiolingual space.

## Supporting information

S1 Table Mean ages and sample sizes in the study groups. (PDF)

S2 Table Post-hoc pairwise tests (Tukey’s honestly significant difference test) of tooth crown diameters (mm), crown area (mm^2^), and reduction indices in the study groups. MD diameter, mesiodistal diameter; BL diameter, buccolingual diameter; Crown area, MD diameter × BL diameter.
(PDF)

S3 Table Multiple regression analysis of the mean crown sizes (MD diameter, BL diameter, and crown area) regressing to karyotypes, Carabelli’s cusp, and the distolingual cusp. MD diameter, mesiodistal diameter; BL diameter, buccolingual diameter; Crown area, MD diameter × BL diameter. ^a^B, partial regression coefficient; ^b^SE.B, standard error; ^c^*β*, standardized partial regression coefficient; ^d^R^2^, R-squared value; ^e^adjusted R-squared value.
(PDF)

S4 Table Multiple regression analysis of the mean crown sizes (MD diameter, BL diameter, and crown area) regressing to the numbers of PAR1 and PAR2 and the expression of Carabelli’s cusp and the distolingual cusp. MD diameter, mesiodistal diameter; BL diameter, buccolingual diameter; Crown area, MD diameter × BL diameter. ^a^B, partial regression coefficient; ^b^SE.B, standard error; ^c^*β*, standardized partial regression coefficient; ^d^R^2^, R-squared value; ^e^Adjusted R-squared value.
(PDF)

S5 Table Logistic regression analysis of Carabelli’s cusp and the distolingual cusp, regressing to karyotypes, crown sizes, and expression of the counter cusp. (PDF)
