# Supplementary material for: Influence of long and short arms of X chromosome on maxillary molar crown morphology
Source: PLoS One. 2018 Nov 15;13(11):e0207070. doi: 10.1371/journal.pone.0207070 (PMC6237344; doi:10.1371/journal.pone.0207070)
Supplement: S1 Table — (PDF) [file pone.0207070.s001.pdf]

S1 Table. Mean ages and sample sizes in the study groups.

Study groups	Karyotypes	n	Mean age (years)	SD (years)	Min. (years)	Max. (years)
Turner female group	46,X,i(Xq)	6	22.9	6.53	16.9	29.2
	45,X/46,XX	16	22.3	9.06	6.8	41.0
	45,X	86	23.0	8.70	5.5	46.4
Control females	46,XX	150	19.0	9.32	5.3	56.0
