# Supplementary material for: Influence of long and short arms of X chromosome on maxillary molar crown morphology
Source: PLoS One. 2018 Nov 15;13(11):e0207070. doi: 10.1371/journal.pone.0207070 (PMC6237344; doi:10.1371/journal.pone.0207070)
Supplement: S2 Table — MD diameter, mesiodistal diameter; BL diameter, buccolingual diameter; Crown area, MD diameter × BL diameter. (PDF) [file pone.0207070.s002.pdf]

S2 Table. Post-hoc pairwise tests (Tukey's honestly significant difference test) of tooth crown diameters (mm), crown area (mm^2), and reduction indices in the study groups.

Measurement	Karyotype (I)	Maxillary first permanent molars (M1)			Maxillary second permanent molars (M2)			Reduction indices		
		Karyotype (J)			Karyotype (J)			Karyotype (J)		
		I	II	III	I	II	III	I	II	III
MD diameter	46,X,i(Xq) (I)									
	45,X (II)	0.529			0.739			0.999		
	45,X/46,XX (III)	0.902	0.844		0.931	0.943		1.000	0.997	
	46,XX	**< 0.001**	**< 0.001**	**< 0.001**	0.087	**0.001**	**0.030**	0.621	**0.015**	0.206
BL diameter	46,X,i(Xq) (I)									
	45,X (II)	0.992			0.790			0.991		
	45,X/46,XX (III)	0.266	**0.042**		0.397	0.539		0.989	1.000	
	46,XX	0.167	**< 0.001**	1.000	0.295	0.086	1.000	0.999	0.966	0.982
Crown area (MD × BL)	46,X,i(Xq) (I)									
	45,X (II)	0.757			0.719			1.000		
	45,X/46,XX (III)	0.532	0.839		0.610	0.956		0.998	0.998	
	46,XX	**0.001**	**< 0.001**	**0.003**	0.087	**0.002**	0.360	0.876	0.096	0.409

MD diameter, mesiodistal diameter; BL diameter, buccolingual diameter; Crown area, MD diameter × BL diameter.
