# Supplementary material for: Influence of long and short arms of X chromosome on maxillary molar crown morphology
Source: PLoS One. 2018 Nov 15;13(11):e0207070. doi: 10.1371/journal.pone.0207070 (PMC6237344; doi:10.1371/journal.pone.0207070)
Supplement: S3 Table — MD diameter, mesiodistal diameter; BL diameter, buccolingual diameter; Crown area, MD diameter × BL diameter. aB, partial regression coefficient; bSE.B, standard error; cβ, standardized partial regression coefficient; dR2, R-squared value; eadjusted R-squared value. (PDF) [file pone.0207070.s003.pdf]

S3 Table. Multiple regression analysis of the mean crown sizes (MD diameter, BL diameter, and crown area) regressing to karyotypes, Carabelli's cusp, and the distolingual cusp.

	Dependent variable	Predictor variables	B [a]	SE.B [b]	β [c]	t-value	*p-value*	R^2 [d]	Adjusted R^2 [e]
M1	MD diameter	Karyotype	0.524	0.059	0.487	8.86	**< 0.001**	0.336	0.327
		Carabelli's cusp	0.176	0.087	0.112	2.014	**0.045**		
		Distolingual cusp	0.548	0.182	0.172	3.011	**0.003**		
	BL diameter	Karyotype	0.150	0.053	0.183	2.848	**0.005**	0.087	0.076
		Carabelli's cusp	0.168	0.077	0.141	2.172	**0.031**		
		Distolingual cusp	0.277	0.162	0.114	1.707	0.089		
	Crown area (MD×BL)	Karyotype	7.305	1.041	0.408	7.021	**< 0.001**	0.260	0.250
		Carabelli's cusp	3.515	1.536	0.134	2.289	**0.023**		
		Distolingual cusp	8.548	3.200	0.161	2.671	**0.008**		
M2	MD diameter	Karyotype	0.289	0.064	0.364	4.544	**< 0.001**	0.161	0.142
		Carabelli's cusp	0.266	0.155	0.144	1.717	0.088		
		Distolingual cusp	0.131	0.105	0.105	1.252	0.213		
	BL diameter	Karyotype	0.163	0.078	0.177	2.080	**0.039**	0.040	0.019
		Carabelli's cusp	0.212	0.191	0.099	1.109	0.269		
		Distolingual cusp	0.045	0.127	0.031	0.355	0.723		
	Crown area (MD×BL)	Karyotype	4.654	1.199	0.318	3.883	**< 0.001**	0.127	0.107
		Carabelli's cusp	4.847	2.925	0.143	1.657	0.100		
		Distolingual cusp	2.042	1.976	0.089	1.034	0.303		

MD diameter, mesiodistal diameter; BL diameter, buccolingual diameter; Crown area, MD diameter × BL diameter. [a]B, partial regression coefficient; [b]SE.B, standard error; [c]β, standardized partial regression coefficient; [d]R^2, R-squared value; [e]adjusted R-squared value.
