# Supplementary material for: Influence of long and short arms of X chromosome on maxillary molar crown morphology
Source: PLoS One. 2018 Nov 15;13(11):e0207070. doi: 10.1371/journal.pone.0207070 (PMC6237344; doi:10.1371/journal.pone.0207070)
Supplement: S4 Table — MD diameter, mesiodistal diameter; BL diameter, buccolingual diameter; Crown area, MD diameter × BL diameter. aB, partial regression coefficient; bSE.B, standard error; cβ, standardized partial regression coefficient; dR2, R-squared value; eAdjusted R-squared value. (PDF) [file pone.0207070.s004.pdf]

S4 Table. Multiple regression analysis of the mean crown sizes (MD diameter, BL diameter, and crown area) regressing to the numbers of PAR1 and PAR2 and the expression of Carabelli's cusp and the distolingual cusp.

		Predictor variables	B [a]	SE.B [b]	*β* [c]	t-value	*p-value*	$R^{2\,d}$	Adjusted $R^{2\,e}$
M1	MD diameter	Number of Xp	0.939	0.145	0.570	6.484	**< 0.001**	0.359	0.348
		Number of Xq	-0.123	0.133	-0.081	-0.925	0.356		
		Carabelli's cusp	0.194	0.086	0.123	2.251	**0.025**		
		Distolingual cusp	0.573	0.178	0.180	3.211	**0.002**		
	BL diameter	Number of Xp	0.394	0.128	0.315	3.091	**0.002**	0.141	0.126
		Number of Xq	-0.023	0.117	-0.020	-0.197	0.844		
		Carabelli's cusp	0.180	0.076	0.151	2.383	**0.018**		
		Distolingual cusp	0.226	0.157	0.093	1.440	0.151		
	Crown area (MD×BL)	Number of Xp	14.346	2.484	0.524	5.774	**< 0.001**	0.319	0.307
		Number of Xq	-1.581	2.284	-0.062	-0.692	0.490		
		Carabelli's cusp	3.859	1.483	0.147	2.602	**0.010**		
		Distolingual cusp	8.281	3.059	0.156	2.707	**0.007**		
M2	MD diameter	Number of Xp	0.526	0.161	0.425	3.261	**0.001**	0.139	0.113
		Number of Xq	-0.151	0.146	-0.134	-1.033	0.303		
		Carabelli's cusp	0.250	0.158	0.136	1.584	0.116		
		Distolingual cusp	0.098	0.107	0.079	0.918	0.360		
	BL diameter	Number of Xp	0.465	0.194	0.323	2.390	**0.018**	0.062	0.034
		Number of Xq	-0.183	0.176	-0.140	-1.038	0.301		
		Carabelli's cusp	0.221	0.190	0.103	1.166	0.246		
		Distolingual cusp	0.015	0.127	0.011	0.120	0.905		
	Crown area (MD×BL)	Number of Xp	9.930	2.987	0.433	3.324	**0.001**	0.136	0.110
		Number of Xq	-3.045	2.701	-0.146	-1.127	0.262		
		Carabelli's cusp	4.762	2.923	0.140	1.629	0.106		
		Distolingual cusp	1.390	1.982	0.060	0.701	0.484		

MD diameter, mesiodistal diameter; BL diameter, buccolingual diameter; Crown area, MD diameter × BL diameter. [a]B, partial regression coefficient; [b]SE.B, standard error; [c]β, standardized partial regression coefficient; [d]R^2, R-squared value; [e]Adjusted R-squared value.
