# Supplementary material for: Influence of long and short arms of X chromosome on maxillary molar crown morphology
Source: PLoS One. 2018 Nov 15;13(11):e0207070. doi: 10.1371/journal.pone.0207070 (PMC6237344; doi:10.1371/journal.pone.0207070)
Supplement: S5 Table — (PDF) [file pone.0207070.s005.pdf]

S5 Table. Logistic regression analysis of Carabelli's cusp and the distolingual cusp, regressing to karyotypes, crown sizes, and expression of the counter cusp.

Dependent variable	Molar	Factors	Odds ratio	95% CI		*p-value*
				Lower	Upper	
Carabelli's cusp	M1	Karyotype (46,X,i(Xq))	0.379	0.059	2.430	0.307
		Karyotype (45,X/46,XX)	1.080	0.272	4.320	0.909
		Karyotype (46,XX)	0.461	0.225	0.940	**0.034**
		Distolingual cusp	20.600	2.520	168.400	**0.005**
		MD diameter	1.490	0.886	2.510	0.132
		BL diameter	1.390	0.758	2.540	0.288
	M2	Karyotype (46,X,i(Xq))	5.890	0.395	87.742	0.198
		Karyotype (45,X/46,XX)	1.329	0.188	9.376	0.775
		Karyotype (46,XX)	0.596	0.167	2.134	0.427
		Distolingual cusp	7.130	2.024	25.118	**0.002**
		MD diameter	2.212	0.710	6.898	0.171
		BL diameter	1.385	0.533	3.600	0.504
Distolingual cusp	M1	Karyotype (46,X,i(Xq))	1.396	0.119	16.311	0.790
		Karyotype (45,X/46,XX)	0.303	0.039	2.357	0.254
		Karyotype (46,XX)	4.249	0.623	28.994	0.140
		Carabelli's cusp	24.993	2.790	223.849	**0.004**
		MD diameter	3.624	1.010	12.998	**0.048**
		BL diameter	3.149	0.699	14.191	0.135
	M2	Karyotype (46,X,i(Xq))	0.635	0.049	8.201	0.728
		Karyotype (45,X/46,XX)	4.475	0.942	21.250	0.059
		Karyotype (46,XX)	1.140	0.475	2.734	0.770
		Carabelli's cusp	7.248	2.046	25.674	**0.002**
		MD diameter	1.832	0.810	4.143	0.146
		BL diameter	0.827	0.433	1.579	0.565
